# Early vs. Late Anticoagulation in Acute Ischemic Stroke for Non-Atrial Fibrillation Indications

**DOI:** 10.21203/rs.3.rs-4578966/v1

**Published:** 2024-07-18

**Authors:** Ming May Zhang, Fady Mousa-Ibrahim, Nicole A. Leshko

**Affiliations:** Northwestern Memorial Hospital; Northwestern Memorial Hospital; Northwestern Memorial Hospital

**Keywords:** ischemic stroke, hemorrhagic stroke, anticoagulation

## Abstract

**Background/Objective:**

In persons whose sole indication for anticoagulation is atrial fibrillation (AF), early therapeutic anticoagulation after acute ischemic stroke (AIS) may decrease ischemic risk without increasing hemorrhagic risk. However, literature to guide anticoagulation timing in patients with a non-AF indication remains extremely limited.

**Methods:**

This retrospective cohort study compared outcomes of early (within ≤4 days of AIS) versus late anticoagulation (5–14 days) for persons with AIS and non-AF indications for anticoagulation. The primary outcome was a composite of intracranial hemorrhage or major extracranial bleeding while on therapeutic anticoagulation, within 30 days of the index event. The main secondary outcome was a composite of major bleeding events while on therapeutic anticoagulation, recurrent AIS, systemic embolism, and all-cause mortality, within 30 days of the index event.

**Results:**

Eighty-one patients were included for analysis, with 65 patients in the early cohort and 16 patients in the late cohort; median time to anticoagulation was 1 day and 7 days, respectively. The most common indication for anticoagulation was deep vein thrombosis. The primary composite outcome occurred in 3 patients (4.6%) in the early cohort and 2 patients (12.5%) in the late cohort (p = 0.255). The secondary composite outcome occurred in 10 patients (15.4%) in the early cohort and 7 patients (43.8%) in the late cohort (p = 0.034). There were no statistical differences in any individual components of the composite outcomes, although recurrent AIS and mortality had numerically higher incidence in the late cohort.

**Conclusions:**

In this retrospective study, early anticoagulation was not associated with increased major bleeding risk, but late anticoagulation was associated with an increased composite risk of major bleeding, thrombotic events, and all-cause mortality, driven by increases in recurrent AIS and mortality. Further studies are warranted to expound on the optimal timing of anticoagulation in this patient population.

## INTRODUCTION

Despite many medical advancements, acute stroke remains a significant clinical problem within the United States (US). An estimated 795,000 Americans per year experience an acute stroke, of which 87% are acute ischemic strokes (AIS). AIS is a leading cause of death in the US and is also associated with severe morbidity, with 87% of stroke survivors reporting long-term physical and/or psychosocial challenges [1].

Persons who present with AIS are at risk of both recurrent ischemic events and hemorrhagic events, including hemorrhagic transformation of infarcted brain tissue. The majority of existing literature regarding the timing of anticoagulation resumption after AIS relates to persons whose sole indication for anticoagulation is non-valvular atrial fibrillation (AF). In this population, a growing body of evidence suggests that early resumption of therapeutic anticoagulation after AIS may decrease ischemic risk without increasing hemorrhagic risk in select patients [2–4]. However, in patients with an indication for anticoagulation other than AF, there is a dearth of literature to guide timing of anticoagulation, making it difficult to balance the hemorrhagic risk of starting anticoagulation versus the thrombotic risk of continuing to withhold therapy [5, 6]. Current guidelines do not comment on the optimal timing to resume or initiate therapeutic anticoagulation in this setting [7, 8].

To our knowledge, only one recent study has assessed anticoagulation timing in a non-AF population. This trial demonstrated that early, low-intensity anticoagulation was associated with decreased thrombotic risk without increasing intracranial hemorrhage (ICH) incidence. However, this study only assessed patients with emergent anticoagulation indications within 3 days of AIS and did not assess extracranial bleeding risk [9]. The purpose of this study is to assess this gap in literature by comparing safety and efficacy outcomes of early therapeutic anticoagulation (initiation within ≤ 4 days of index ischemic stroke) vs. late anticoagulation (initiation within 5–14 days) in AIS for both chronic and acute indications other than AF.

## METHODS

### Study Design, Setting, and Population:

This was a retrospective, observational single-site cohort study conducted at Northwestern Memorial Hospital. Patients were included if they were ≥18 years old, admitted to Northwestern Memorial Hospital between January 1, 2022 to December 31, 2022, had a diagnosis of AIS not secondary to AF during their admission, and had an indication for therapeutic anticoagulation other than AF. Patients were excluded if they met any of the following exclusion criteria: 1) Anticoagulation not initiated within 14 days of AIS, 2) Presence of a ventricular assist device, 3) Receipt of decompressive craniotomy during the admission, 4) AIS etiology warranting immediate anticoagulation (e.g. vertebral artery dissection), 5) Decision to transition to hospice or comfort-focused care during the admission, or 6) Pregnancy, incarceration, or enrollment in a clinical trial during the study time period.

This study was approved by the Northwestern University Institutional Review Board and was performed in accordance with the ethical standards as laid down in the 1964 Declaration of Helsinki and its later amendments.

### Data Collection:

A preliminary informatics report identified patients admitted within the defined time frame who met initial inclusion criteria; patients were then manually screened for exclusion criteria. For patients who met full inclusion criteria, data for baseline characteristics and primary and secondary outcomes were manually collected via chart review. Data were stored in a Research Electronic Data Capture database.

### Outcomes and Clinical Definitions:

Patients were counted within the early anticoagulation cohort if therapeutic anticoagulation was initiated within ≤4 days of the index ischemic stroke and the late cohort if initiated within 5–14 days, extrapolated from previously reported thresholds in the AF-related AIS literature and guidelines [2,7,8]. The primary outcome was a composite of major bleeding events while on therapeutic anticoagulation, defined as ICH or major extracranial bleeding within 30 days of the index event. The main secondary outcome was a composite of major bleeding events while on therapeutic anticoagulation, recurrent AIS, systemic embolism, and all-cause mortality within 30 days of the index event. Other outcomes, assessed at 30 days of the index event unless otherwise indicated, included individual components of the composite outcomes and time to occurrence for each initial event, clinically relevant non-major bleeding while on therapeutic anticoagulation and time to first non-major bleeding event, and modified Rankin Scale (mRS) at discharge, when available.

Diagnosis of index AIS was determined based on chart review of neuroimaging (computed tomography [CT] or magnetic resonance imaging [MRI]) documented within the electronic health record (EHR), with the date of the first image confirming AIS counted as the date of AIS onset. AIS etiologies were determined by review of clinical documentation; two reviewers independently assigned a correlating Trial of Org 10172 in Acute Stroke Treatment (TOAST) category, with discrepancies resolved via a third reviewer [10]. Recurrent AIS was defined as new acute infarcts or worsening infarct burden confirmed on neuroimaging, occurring >24 hours after the index AIS. Systemic embolism was defined as acute vascular occlusion of any extremity or organ confirmed by imaging.

ICHs, including hemorrhagic transformation of the index stroke, were counted toward the outcome if they were defined on neuroimaging and occurred while on therapeutic anticoagulation as defined below. For patients who experienced hemorrhagic transformation prior to receiving anticoagulation, ICHs were only counted towards the outcome if neuroimaging confirmed a new or expanding ICH after initiation of anticoagulation. Major extracranial bleeding was defined per International Society for Thrombosis and Hemostasis (ISTH) criteria: Fatal bleeding; and/or symptomatic bleeding in a critical area or organ; and/or bleeding causing a fall in hemoglobin levels of >/=1.24 mmol/L or leading to a transfusion of >/=2 units of whole blood or red cells) [11]. Clinically relevant non-major bleeding was also defined per ISTH criteria: Hemorrhage that does not fit the criteria for the ISTH definition of major bleeding but does meet at least one of the following criteria: 1) requiring medical intervention by a healthcare professional, 2) leading to hospitalization or increased level of care, or 3) prompting a face-to-face evaluation) [11]. Patients were considered to be on therapeutic anticoagulation after at least 1 dose of therapeutic enoxaparin or a direct-acting oral anticoagulant (DOAC), after international normalized ratio (INR) was within therapeutic range for warfarin, or after activated partial thromboplastin time (aPTT) or anti-factor Xa (anti-Xa) was within therapeutic range for heparin infusion.

The mRS score at discharge was determined by review of discharge summaries within the EHR. Only documented mRS scores, when available, were counted toward the outcome; no mRS scores were retrospectively calculated by the members of this study.

### Stroke Characterization on Neuroimaging:

A neurologist reviewed initial neuroimaging of the index AIS to determine infarct volume and supratentorial versus infratentorial involvement. If both CT and MRI imaging were available, MRI imaging was utilized. If multiple MRIs were available, the first post-AIS MRI was utilized. Infarct volumes <1 mL were not quantitatively assessed, but infarct volumes ≥1 mL were measured with the ellipsoid formula ABC/2. This formula was used to calculate the largest infarct volume visualized on imaging. Slices of the lesion with a volume greater than 75% were multiplied by 1. For slices with a lesion volume between 25% and 75%, the slice thickness was multiplied by 0.5. Slices with volume less than 25% of the largest lesion volume were not counted in the z axis [12].

### Statistical Analysis:

Data were summarized using means and standard deviations for parametric continuous data, medians and interquartile ranges (IQRs) for ordinal or non-parametric continuous data, and frequencies and percentages for nominal data. Baseline characteristics and outcomes were compared between cohorts using the student T-test for parametric continuous data, the Mann-Whitney U test for non-parametric continuous data or ordinal data, and Chi-square or Fisher’s exact tests for categorical data. *p* values ≤ 0.05 were considered statistically significant. All analyses were performed using SPSS version 29.0 software.

## RESULTS

### Study Population:

**Figure 1:**
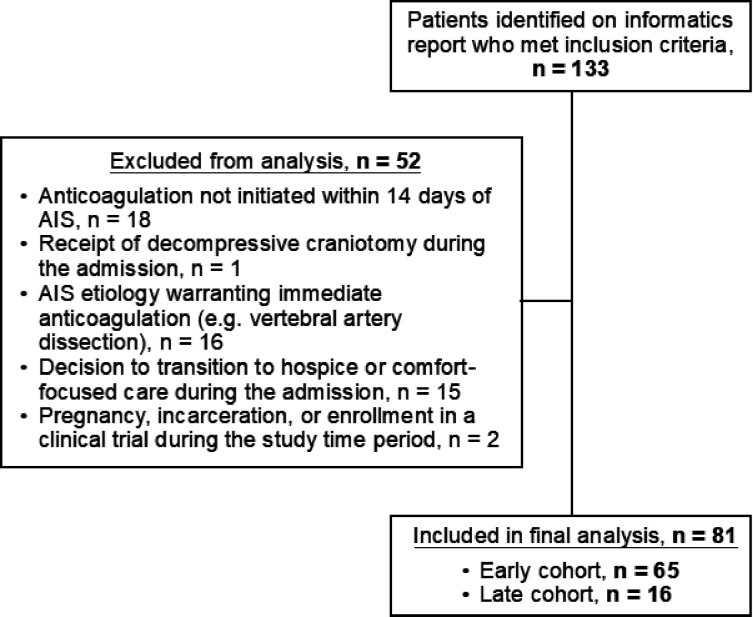
Application of Inclusion and Exclusion Criteria Abbreviations: AIS: acute ischemic stroke Application of inclusion and exclusion criteria for selection of final cohort

A total of 133 patients meeting inclusion criteria were identified on an initial informatics report. Of these patients, 52 met exclusion criteria, resulting in a final cohort of 81 patients: 65 patients in the early cohort and 16 patients in the late cohort (Figure 1). Baseline characteristics are summarized in Table 1. Mean age was 64 years, and past medical history was similar in both cohorts; notable medical history within the total cohort included 6 patients (7.4%) with AF, 16 patients (19.8%) with prior AIS, and 2 patients (2.5%) with prior ICH. There was no significant difference in the proportion of patients who utilized anticoagulant or antiplatelet therapy prior to the index stroke, although there was a statistical difference in the distribution of the anticoagulant agent used. Within the whole cohort, the most common indication for anticoagulation was deep vein thrombosis (DVT), followed by pulmonary embolism (PE), hypercoagulopathic disease state (e.g. antiphospholipid syndrome, Factor V Leiden, JAK2 mutation, or hypercoagulability of malignancy), intracardiac thrombus, embolic stroke of unknown etiology (ESUS), bioprosthetic valve, or another indication. No patients had a mechanical valve as their indication for anticoagulation. If patients had multiple indications for anticoagulation, all indications were counted.

Median initial National Institutes of Health Stroke Scale (NIHSS) score was 3 in the early cohort compared to 14 in the late cohort (p = 0.007). Based on the TOAST classification system, the most common stroke etiology within the total cohort was stroke of undetermined etiology (33.3%) and stroke of other determined etiology (33.3%), followed by cardioembolic stroke (25.9%), small-vessel occlusion (3.7%), and large-artery atherosclerosis (3.7%). Among patients with a stroke of other determined etiology, the most common etiology was a hypercoagulopathic disease state. MR diffusion-weighted imaging (DWI) was available for 77 patients; the other 4 had only CT imaging available. Thirty-seven patients (56.9%) in the early cohort had a measurable infarct volume (i.e. ≥1 mL), compared to 12 patients (75.0%) in the late cohort (p = 0.185), with median volumes of 4.5 mL (IQR 2.2, 8.8) and 13.3 mL (IQR 3.9, 38.3), respectively (p = 0.018). Infratentorial infarcts were seen in 16 patients (24.6%) in the early cohort and 1 patient (6.3%) in the late cohort (p = 0.230). Six patients (9.2%) in the early cohort and 2 patients (12.5%) in the late cohort received thrombolysis (p = 0.654). Endovascular thrombectomy (EVT) was performed in 5 patients (7.7%) in the early cohort and 6 patients (37.5%) in the late cohort (p = 0.006). Hemorrhagic transformation, prior to starting anticoagulation, occurred in 3 patients [4.6%] in the early cohort versus 3 patients [18.8%] in the late cohort (p = 0.088).

Median time to therapeutic anticoagulation was 1 day in the early cohort and 7 days in the late cohort (p <0.01); distribution of anticoagulant agents was similar between cohorts, with apixaban being the most commonly utilized anticoagulant. Twenty-four (36.9%) patients in the early cohort received DVT prophylaxis prior to the initiation of therapeutic anticoagulation, compared to 15 patients (93.8%) in the late cohort (p < 0.001)

### Study Outcomes:

Full study outcomes are summarized in Table 2. The primary composite outcome of ICH or major extracranial bleeding occurred in 3 patients (4.6%) in the early cohort and 2 patients (12.5%) in the late cohort (p = 0.255). ICH occurred in 1 patient (1.5%) in the early cohort and 1 patient (6.3%) in the late cohort (p = 0.358), and major extracranial bleeding occurred in 3 patients (4.6%) in the early cohort and 1 patient (6.3%) in the late cohort (p = 1.000). The secondary composite outcome of ICH, major extracranial bleeding, recurrent AIS, systemic embolism, and all-cause mortality occurred in 10 patients (15.4%) in the early cohort compared to 7 patients (43.8%) in the late cohort (odds ratio [OR] 0.23, 95% confidence interval [CI] 0.07–0.77); this difference was primarily driven by a numerically higher incidence of recurrent AIS (early cohort n = 5 [7.7%]; late cohort n = 3 [18.8%]; OR 0.36, 95% CI 0.08–1.70) and mortality (early cohort n = 3 [4.6%]; late cohort n = 3 [18.8%]; OR 0.21, 95% CI 0.04–1.16) in the late cohort. Median discharge mRS, only collected in a subset of patients who had this outcome documented, was 3 in the early cohort and 4 in the late cohort (p = 0.051). Median length of stay was 6 days in the early cohort and 14 days in the late cohort (p < 0.01).

In a subgroup of patients who experienced hemorrhagic transformation prior to initiation of anticoagulation, the primary composite outcome occurred in 0 of 3 patients (0.0%) in the early cohort and 1 of 3 patients (33.3%) in the late group, and the secondary composite outcome occurred in 0 of 3 patients (0.0%) in the early cohort and 1 of 3 patients (33.3%) in the late cohort.

## DISCUSSION

In this retrospective cohort study, early anticoagulation for non-AF indications after AIS was not associated with increased major bleeding risk compared to late anticoagulation. However, late anticoagulation was associated with an increased composite risk of major bleeding, thrombotic events, and all-cause mortality, driven by increases in recurrent AIS and mortality. Our findings are consistent with Jumah et al., who studied a non-AF population with emergent indications for anticoagulation and found that early anticoagulation (within 3 days) correlated with decreased thrombotic risk but not increased ICH incidence. The ICH and overall thromboembolic rates reported in this study are also congruent with those observed by Jumah et al., although our population experienced higher rates of recurrent AIS and lower rates of VTE [9].

Our findings additionally correlate to those of the TIMING and ELAN trials, which studied post-AIS patients whose indication for anticoagulation was AF. These trials found that early anticoagulation was non-inferior to late anticoagulation for primary composite outcomes including ICH, recurrent AIS, and mortality; additionally, early anticoagulation was associated with numerically decreased AIS without increasing ICH incidence [2,3]. As whole, our cohort experienced higher rates of both major bleeding and major thrombotic outcomes than those seen in ELAN and TIMING (Table 3). One potential explanation for this difference is that our cohort may have been more medically complex, as evidenced by its variety of stroke etiologies and indications for anticoagulation and larger stroke burden in the late initiation cohort. Additionally, due to the limited size of our population, a low number of absolute event rates may appear to have a higher relative frequency than would be seen in the larger cohorts studied in the AF population. Further research is needed to better elucidate the rates of bleeding and thrombotic events in a non-AF post-AIS population.

Therapeutic anticoagulation was initiated relatively quickly in our study, with a median time to anticoagulation of 1 day (IQR 0, 4) in the total cohort. Current guidelines do not comment on timing of anticoagulation initiation for this patient population, but in the setting of AF, the American Stroke Association recommends anticoagulation 4–14 days after AIS, and the European Stroke Organization recommends anticoagulation 3–14 days after AIS, depending on infarct size [7,8]. In the TIMING trial, median time to anticoagulation was 3 days in the early cohort and 5 days in the late cohort [2]. Several factors may have contributed to the rapid initiation of anticoagulation in our study. First, the majority of our population experienced minor strokes with a low NIHSS, small infarct volume, and mostly supratentorial involvement; therefore, clinicians may have started anticoagulation sooner due to a low risk of hemorrhagic conversion. Second, most strokes in our cohort were caused by etiologies for which anticoagulation would likely be beneficial and reduce the risk of recurrent AIS (e.g. hypercoagulopathic disease states, ESUS, cardioembolic strokes), which might similarly prompt clinicians to initiate anticoagulation more rapidly.

This study has a number of limitations, which should be considered in the interpretation of its findings. First, given the retrospective nature of this study, there were multiple measured and unmeasured confounding factors that may have affected our outcomes. For instance, median NIHSS and stroke volume were significantly higher in the late cohort, suggesting a more severe stroke presentation that could have contributed to an increased outcome event rate. The late cohort was also more likely to receive an EVT and had a numerically higher incidence of hemorrhagic transformation prior to anticoagulation, both of which may have increased ICH risk. Additionally, the late cohort had a statistically longer LOS; in addition to suggesting that these patients were more medically complex and thus predisposed to experiencing an outcome event, this could also have led to information bias since patients in the inpatient setting are more likely to have events detected compared to discharged patients. Second, our total population size was limited to 81 patients, of which only 16 patients were included in the late cohort. This may decrease the internal validity of our findings. Third, given a median NIHSS of 4 (IQR 1, 9) and median infarct volume (for measurable infarcts) of 4.6 mL (IQR 2.6, 11.9) in our total cohort, we cannot draw strong conclusions regarding bleeding and thrombotic risk of early anticoagulation for more severe AIS presentations.

Despite these limitations, this study explores an important clinical question for which published literature remains extremely scarce. The major strength of this study is the successful creation of the population of interest: post-AIS patients with both acute and chronic non-AF indications for AC, as evidenced by the 7.4% incidence of AF in our total cohort, compared to the AF-only cohorts studied in the published literature [2,3]. Our findings provide crucial initial information regarding the benefits and risks of early versus late anticoagulation in this population and justify the need for larger observational studies, as well as randomized controlled trials exploring optimal anticoagulation timing. Particular subpopulations of interest include patients with more severe AIS presentations and patients with stroke etiologies that would not directly benefit from anticoagulation (e.g. small-vessel occlusion, large-artery atherosclerosis), as these patients may have a differing balance of bleeding and thrombotic risk.

## CONCLUSIONS

In this limited retrospective study of post-AIS patients with a non-AF indication for anticoagulation, early anticoagulation (≤ 4 days) was not associated with increased major bleeding risk compared to late anticoagulation (5–14 days), but late anticoagulation was associated with an increased composite risk of major bleeding, thrombotic events, and all-cause mortality. Further studies are warranted to expound on the optimal timing of therapeutic anticoagulation in this patient population.

## Figures and Tables

**Table 1.** Baseline Characteristics

	Early group (N = 65)	Late group (N = 16)	Total (N = 81)	p value
**CLINICAL CHARACTERISTICS**				
Age (years), mean (SD)	63 (14)	68 (13)	64 (14)	0.201
Gender, n (%)				0.956
Female	32 (49.2%)	8 (50.0%)	40 (49.4%)	
Male	33 (50.8%)	8 (50.0%)	41 (50.6%)	
SCr (mg/dL), median (IQR)	0.93 (0.72,1.3)	0.94 (0.79,1.76)	0.93 (0.73,1.34)	0.510
Weight (kg), median (IQR)	76.2 (69.9,92.0)	76.7 (69.9,86.2)	76.2 (69.9,90.5)	0.709
PMH, n (%)				
HF	10 (15.4%)	1 (6.3%)	11 (13.6%)	0.684
HTN	28 (43.1%)	10 (62.5%)	38 (46.9%)	0.163
DM	13 (20.0%)	2 (12.5%)	15 (18.5%)	0.723
CAD	9 (13.8%)	2 (12.5%)	11 (13.6%)	1.000
Atrial fibrillation, n (%)	4 (6.2%)	2 (12.5%)	6 (7.4%)	0.338
CHA2DS2-VASc, median (IQR)	5 (4, 6)	6 (--)	6 (4, 6)	1.000
AIS	13 (20.0%)	3 (18.8%)	16 (19.8%)	1.000
TIA	5 (7.7%)	0 (0.0%)	5 (6.2%)	0.577
ICH	1 (1.5%)	1 (6.3%)	2 (2.5%)	0.358
Pre-stroke AC use, n (%)	31 (47.7%)	5 (31.3%)	36 (44.4%)	0.236
Apixaban	23 (74.2%)	3 (60.0%)	26 (72.2%)	**0.047**
Rivaroxaban	5 (16.1%)	0 (0.0%)	5 (13.9%)	
Warfarin	1 (3.2%)	1 (20.0%)	2 (5.6%)	
Enoxaparin	2 (6.5%)	0 (0.0%)	2 (5.6%)	
UFH	0 (0.0%)	1 (20.0%)	1 (2.8%)	
Pre-stroke antiplatelet use, n (%)	21 (32.3%)	8 (50.0%)	29 (35.8%)	0.186
Aspirin	20 (30.8%)	7 (43.8%)	27 (33.3%)	0.324
Clopidogrel	3 (4.6%)	1 (6.3%)	4 (4.9%)	1.000
Prasugrel	0 (0.0%)	0 (0.0%)	0 (0.0%)	--
Ticagrelor	1 (1.5%)	0 (0.0%)	1 (1.2%)	1.000
Dipyridamole	1 (1.5%)	0 (0.0%)	1 (1.2%)	1.000
Baseline mRS^a^, median (IQR)	1 (0, 3)	3 (1, 4)	2 (0, 3)	0.359
**INDICATIONS FOR ANTICOAGULATION**				
DVT, n (%)	24 (36.9%)	9 (56.3%)	33 (40.7%)	0.159
Timing				0.178
<3 months before AIS	13 (54.2%)	8 (88.9%)	21 (63.6%)	
3–12 months before AIS	1 (4.2%)	0 (0.0%)	1 (3.0%)	
>12 months before AIS	10 (41.7%)	1 (11.1%)	11 (33.3%)	
Proximal?	10 (41.7%)	3 (33.3%)	13 (39.4%)	0.325
PE, n (%)	17 (26.2%)	4 (25.0%)	21 (25.9%)	1.000
Timing				0.462
<3 months before AIS	12 (70.6%)	4 (100.0%)	16 (76.2%)	
3–12 months before AIS	2 (11.8%)	0 (0.0%)	2 (9.5%)	
>12 months before AIS	3 (17.6%	0 (0.0%)	3 (14.3%)	
Severity				0.066
Massive	0 (0.0%)	0 (0.0%)	0 (0.0%)	
Submassive	6 (35.3%)	0 (0.0%)	6 (28.6%)	
Non-massive	6 (35.3%)	4 (100.0%)	10 (47.6%)	
Unknown	5 (29.4%)	0 (0.0%)	5 (23.8%)	
Hypercoagulopathic disease state (e.g. APLS, FVL, JAK2 mutation, hypercoagulability of malignancy), n (%)	14 (21.5%)	3 (18.8%)	17 (21.0%)	1.000
Intracardiac thrombus, n (%)	6 (9.2%)	4 (25.0%)	10 (12.3%)	0.102
ESUS, n (%)	10 (15.4%)	0 (0.0%)	10 (12.3%)	0.198
Bioprosthetic valve, n (%)	2 (3.1%)	1 (6.3%)	3 (3.7%)	0.488
Aortic	2 (100.0%)	1 (100.0%)	3 (100.0%)	--
Implanted within ≤3 months	2 (100.0%)	1 (100.0%)	3 (100.0%)	--
Other, n (%)	10 (15.4%)	3 (18.8%)	13 (16.0%)	0.714
**STROKE CHARACTERISTICS**				
Initial NIHSS^b^, median (IQR)	3 (1, 8)	14 (5, 20)	4 (1, 9)	**0.007**
Stroke etiology (TOAST classification), n (%)				0.634
Stroke of undetermined etiology	23 (35.4%)	4 (25.0%)	27 (33.3%)	
Stroke of other determined etiology	22 (33.8%)	5 (31.3%)	27 (33.3%)	
Hypercoagulopathic disease state (e.g. APLS, FVL, JAK2 mutation, hypercoagulability of malignancy)	19 (86.4%)	4 (80.0%)	23 (85.2%)	1.000
Cardioembolic stroke	15 (23.1%)	6 (37.5%)	21 (25.9%)	
Infective endocarditis	2 (13.3%)	0 (0.0%)	2 (9.5%)	1.000
Small-vessel occlusion	3 (4.6%)	0 (0.0%)	3 (3.7%)	
Large-artery atherosclerosis	2 (3.1%)	1 (6.3%)	3 (3.7%)	
Multiple acute infarcts, n (%)	52 (80.0%)	11 (68.8%)	63 (77.8%)	0.332
Patients with infarct volume(s) ≥1 mL, n (%)	37 (56.9%)	12 (75.0%)	49 (60.5%)	0.185
Infarct volume (mL) of infarcts ≥1 mL, median (IQR)	4.5 (2.2,8.8)	13.3 (3.9,38.3)	4.6 (2.6,11.9)	**0.018**
Location of infarct(s), n (%)				0.230
Supratentorial	49 (75.4%)	15 (93.8%)	64 (79.0%)	
Infratentorial	8 (12.3%)	1 (6.3%)	9 (11.1%)	
Both supra- and infratentorial	8 (12.3%)	0 (0.0%)	8 (9.9%)	
Receipt of fibrinolytic, n (%)	6 (9.2%)	2 (12.5%)	8 (9.9%)	0.654
Alteplase	5 (83.3%)	2 (100.0%)	7 (87.5%	1.000
Unknown	1 (16.7%)	0 (0.0%)	1 (12.5%)	
Endovascular thrombectomy, n (%)	5 (7.7%)	6 (37.5%)	11 (13.6%)	**0.006**
TICI score of 2b, 2c, or 3	5 (100.0%)	6 (100.0%)	11 (100.0%)	--
TICI 2b	2 (40.0%)	3 (50.0%)	5 (45.5%)	0.792
TICI 2c	0 (0.0%)	2 (33.3%)	2 (18.2%)	
TICI 3	3 (60.0%	1 (16.7%)	4 (36.4%)	
Hemorrhagic transformation prior to starting anticoagulation, n (%)	3 (4.6%)	3 (18.8%)	5 (6.2%)	0.088
**POST-STROKE ANTICOAGULATION MANAGEMENT**				
Time to initiation of therapeutic AC (days post-AIS), median (IQR)	1 (0, 2)	7 (6, 9)	1 (0, 4)	**<0.01**
DVT prophylaxis prior to therapeutic AC, n (%)	24 (36.9%)	15 (93.8%)	39 (48.1%)	**<0.001**
Bridging with parenteral therapeutic AC, n (%)	9 (13.8%)	2 (12.5%)	11 (13.6%)	1.000
UFH infusion, goal antiXa 0.3–0.7	5 (55.6%)	1 (50.0%)	6 (54.5%)	0.727
Enoxaparin	4 (44.4%)	1 (50.0%)	5 (45.5%)	
Maintenance AC agent, n (%)				0.805
Apixaban	33 (50.8%)	10 (62.5%)	43 (53.1%)	
Enoxaparin	14 (21.5%)	3 (18.8%)	17 (21.0%)	
Warfarin	13 (20.0%)	3 (18.8%)	16 (19.8%)	
Rivaroxaban	4 (6.2%)	0 (0.0%)	4 (4.9%)	
Dabigatran	1 (1.5%)	0 (0.0%)	1 (1.2%)	

a. Baseline mRS: N = 37 for early group, N = 4 for late group, N = 41 for total

b. NIHSS: N = 61 for early group, N = 14 for late group, N = 75 for total

Abbreviations: AC: anticoagulation, AIS: acute ischemic stroke, APLS: antiphospholipid syndrome, CAD: coronary artery disease, DM: diabetes mellitus, DVT: deep vein thrombosis, ESUS: embolic stroke of undetermined source, FVL: factor V Leiden, HF: heart failure, HTN: hypertension, ICH: intracranial hemorrhage, IQR: interquartile ratio, mRS: modified Rankin scale, NIHSS: National Institutes of Health Stroke Scale, PE: pulmonary embolism, SCr: serum creatinine, SD: standard deviation, TIA: transient ischemic attack, TOAST: Trial of Org 10172 in Acute Stroke Treatment, UFH: unfractionated heparin

**Table 2.** Primary and Secondary Outcomes^a^

	Early group (N = 65)	Late group (N = 16)	Total (N = 81)	p value
**PRIMARY OUTCOME**				
Composite of ICH and major extracranial bleeding, n (%)	3 (4.6%)	2 (12.5%)	5 (6.2%)	0.255
**SECONDARY OUTCOMES**				
Composite of ICH, major extracranial bleeding, recurrent AIS, systemic embolism, and all-cause mortality, n (%)	10 (15.4%)	7 (43.8%)	17 (21.0%)	**0.034**
ICH, n (%)	1 (1.5%)	1 (6.3%)	2 (2.5%)	0.358
Hemorrhagic transformation?	1 (100.0%)	1 (100.0%)	2 (100.0%)	--
Location				1.000
Subarachnoid	0 (0.0%)	1 (100.0%)	1 (50.0%)	
Intraparenchymal	1 (100.0%)	0 (0.0%)	1 (50.0%)	
Volume (mL)	1	<1	--	--
Time to occurrence (days post-AIS), median (IQR)	5 (--)	7 (--)	6 (--)	1.000
Major extracranial bleeding, n (%)	3 (4.6%)	1 (6.3%)	4 (4.9%)	1.000
Time to occurrence (days post-AIS), median (IQR)	18 (--)	14 (--)	16 (13, 25)	1.000
Clinically relevant non-major bleeding, n (%)	4 (6.2%)	3 (18.8%)	7 (8.6%)	0.135
Time to occurrence (days post-AIS), median (IQR)	18 (7, 26)	10 (--)	10 (9, 26)	0.629
Recurrent AIS, n (%)	5 (7.7%)	3 (18.8%)	8 (9.9%)	0.189
Time to occurrence (days post-AIS), mean (SD)	12 (9)	6 (4)	10 (8)	0.337
Systemic embolism, n (%)	4 (6.2%)	2 (12.5%)	6 (7.4%)	0.338
Time to occurrence (days post-AIS), median (IQR)	9 (3, 24)	4 (--)	6 (3, 15)	0.533
All-cause mortality, n (%)	3 (4.6%)	3 (18.8%)	6 (7.4%)	0.088
Discharge mRS^b^, median (IQR)	3 (1,4)	4 (3, 4)	3 (1,4)	0.051
Hospital LOS (days), median (IQR)	6 (4, 11)	14 (7, 21)	7 (4, 13)	**<0.01**

a. All outcomes measured at 30 days except for mRS (which was measured at discharge) and length of stay

b. Discharge mRS: N = 40 for early group, N = 6 for late group, N = 46 for total

Abbreviations: AIS: acute ischemic stroke, ICH: intracranial hemorrhage, IQR: interquartile ratio, LOS: length of stay, mRS: modified Rankin scale, SD: standard deviation

**Table 3.** Comparative Outcome Rates in Previous Literature [2,3]

	Current study	TIMING^a^	ELAN^b^

Total N	81	888	2013

ICH	Early: 1.5%	Early: 0.4%	Early: 0.2%
	Late: 6.3%	Late: 0.2%	Late: 0.2%

Major extracranial bleed	Early: 6.3%	Early: 1.1%	Early: 0.3%
	Late: 4.6%	Late: 0.5%	Late: 0.5%

Recurrent AIS	Early: 7.7%	Early: 3.1%	Early: 1.4%
	Late: 18.8%	Late: 4.6%	Late: 2.5%

Systemic embolism	Early: 6.2%	--	Early: 0.4%
	Late: 12.5%		Late: 0.9%

a. ICH and major extracranial bleed rates calculated based on textual description of major bleeding events

b. Only symptomatic ICH reported

Abbreviations: AIS: acute ischemic stroke, ICH: intracranial hemorrhage
